# Genetic Diversity of Hydro Priming Effects on Rice Seed Emergence and Subsequent Growth under Different Moisture Conditions

**DOI:** 10.3390/genes11090994

**Published:** 2020-08-25

**Authors:** Yoshihiro Nakao, Chiharu Sone, Jun-Ichi Sakagami

**Affiliations:** 1 Course of Science of Bioresource Production, The United Graduate School of Agricultural Sciences, Kagoshima University, 1-21-24 Korimoto, Kagoshima 890-0065, Japan; nakao.yoshi.kagoshima@gmail.com; 2 Department of Biological production, Faculty of Bioresource Sciences, Akita Prefectural University, 241-438 Kaidobata-Nishi Shimoshinjo Nakano, Akita 010-0195, Japan; ccsone@akita-pu.ac.jp; 3 Department of Agricultural Sciences and Natural Resources, Faculty of Agriculture, Kagoshima University, 1-21-24 Korimoto, Kagoshima 890-0065, Japan

**Keywords:** germination, cultivar, genotype, drought, Africa

## Abstract

Seed priming refers to seed enhancement methods that stimulate seed metabolism. This study evaluated the genetic diversity of hydro priming efficacy in 27 different genotypes of rice under dry to wet soil moisture conditions. The genotypes included 21 genotypes of *Oryza sativa*, five genotypes of *Oryza glaberrima*, and one genotype of NERICA (New Rice for Africa). The treated rice seeds were sown in plastic boxes under four soil moisture conditions (5%, 10%, 15%, and 20% (*w*/*w*)). The genotypes were categorized into six groups based on growth parameters using hierarchical cluster analysis. Furthermore, emergence properties were investigated by using principal component analysis based on the mean emergence time of control and primed seeds. Seed priming enhanced growth performance under the moderate dry conditions of 10% and 15% soil moisture. Meanwhile, priming efficacy was low in water stress conditions of 5% and 20% soil moisture. There were wide-ranging genotypic differences of priming efficacy under 20% soil moisture condition. Our findings indicate that the anaerobic-tolerant genotypes tend to exhibit priming efficacy under high soil moisture conditions. Furthermore, one group included all upland genotypes of *Oryza sativa*. This group originally adapted to 10% and 15% of dry conditions, and seed priming improved their features greatly.

## 1. Introduction

In recent years, the growth in demand for rice has been faster in sub-Saharan Africa (SSA) than anywhere else in the world [1]. Although West Africa is still the hub of rice production in SSA, there has been a significant increase in the shortfall of rice production as the rate of consumption increases well above the rate of production [2]. Rice cultivation in SSA is conducted in four ecosystems: dryland, rainfed wetland, deep water and mangrove swamps, and irrigated wetland, with the percentages of the total cultivated area being 38%, 33%, 9%, and 20%, respectively [1]. In West Africa, 75% of the total rice production from 1993 to 2003 came from upland, hydromorphic, and lowland ecosystems, with approximately 25% coming from irrigated fields [3]. Thus, in Africa, most rice is cultivated on rainfed uplands and lowlands without an irrigation system. Balasubramanian et al. (2007) [1] reported that the boundary between wetland and dryland is often gradual on the lower slope. In upland systems, the average rice yield is approximately 1 t ha^−1^, and, in rainfed lowlands, the rice yield depends on the degree of water control and varies from 1 to 3 t ha^−1^ [3]. As is well-known, direct seeding is a standard method of rice sowing in upland rice cultivation in Africa; however, stand establishment of dry-seeded rice can be poor due to erratic rainfall and frequent drought after seeding [4]. Farooq et al. (2011) [5] reported that crop establishment is the key factor in the subsequent growth, development, and yield of direct-seeded rice. Shortage of rainfall results in poor initial growth and subsequent low yield of upland rice. Therefore, a key technology is required to improve plant emergence and establishment under unstable soil moisture conditions. Seed priming is a well-known seed enhancement method. In rice, seed priming improves seed germination, plant emergence, subsequent growth, and yield [6,7]. As reported by Khan (1992) [8], the term “priming of seed” was coined in the early 1970s. Taylor et al. (1998) [9] defined seed enhancement as a post-harvest treatment that improves germination or seedling growth or facilitates the delivery of seeds and other materials required during sowing. Priming alters enzyme activities and promotes the mobilization of sugars [6,10]. Several seed priming methods have been studied, e.g., hydro priming, osmotic-priming by using CaCl_2_ or KCl, and polyethylene glycol and ascorbate-priming [11,12]. As reported by Soltani and Soltani (2015) [13], hydro priming was recommended because of its affordability and enhancement of seed germination, seedling emergence, and crop yield. Thus, several studies about hydro priming techniques have been carried out in Africa [14,15,16]. Recent findings show that the efficacy of seed priming depends on soil moisture conditions [7] and seed genotypes [17,18]. The current study focused on the different growth patterns of primed seed under several different soil moisture conditions. The aim was to assess the genetic diversity of the hydro priming efficacy on rice, including local cultivars from Ghana, under a range of soil moisture conditions.

## 2. Materials and Methods

Twenty-seven rice genotypes, including *Oryza sativa* L., *O. glaberrima* Steud. and interspecific hybrids between *O. sativa* and *O. glaberrima*, known as NERICA (New Rice for Africa), were used in the experiment (Table 1). To break seed dormancy, seeds were exposed to a temperature of 48 °C for 10 days in an oven. Low-quality seeds (specific density < 1.13) were not used. Selected seeds were washed in water and air-dried. The hydro priming treatment consisted of soaking seeds in tap water at 28 °C for incubation and air-drying them to the original dry seed weight. The soaking time was determined in a pre-germination test using a petri dish with filter paper saturated with distilled water at 28 °C for incubation. Control seeds were not treated as primed seeds. Ten seeds were sown in triplicate, and the number of germinated seeds was counted every 6 h. The soaking time was decided as being 6 h before the time when the first germination was observed. A germination test for comparing control and priming was conducted in the same manner as the pre-germination test. We calculated the time to achieve 50% germination (G50) and the mean germination time (MGT) according to the following formula.

G50 = *t_i_* + (N/2 − *n_i_*) * (*t_j_* − *t_i_*)/(*n_j_* − *n_i_*)

where N is the final number of seeds that germinated, and n_i_ and n_j_ are the cumulative number of seeds germinated, as determined by adjacent counts at times *t_i_* and *t_j_*, respectively, when *n_i_* < N/2 < *n_j_* [7].

$$\bar{t}\;(\mathrm{MGT})\;=\;\sum_{i=1}^{k}\left(n_{i}*t_{i}\right)/\sum_{i=1}^{k}n_{i}$$

where *t_i_* is the time from the start of the experiment to the *i*th observation (day or hour); *n_i_* is the number of seeds germinated in time *i* (not the accumulated number, but the number corresponding to the *i*th observation), and *k* is the last time of germination [19].

For the cultivation experiment, we used plastic pots (depth 270 mm, width 540 mm, and height 60 mm) in an experimental room (mean temperature 29.0 °C and mean humidity 73.9%.) The pots were filled with soil taken from the White Volta flood plain in the northern region of Ghana (lat. 9°06′ N, long. 1°09′ W) [20]. Regarding soil texture, clay, silt, and sand was 7.0%, 29.4%, and 63.6% respectively. N and P content was 12.7 and 3.8 mg kg^−1^, respectively. Exchange cation of Ca, K, Mg, and Na was 0.79, 0.09, 0.22, and 0.02 mol_c_ kg^−1^, respectively. Soil pH was 6.0. The soil was air-dried and gravel was removed by using a sieve. Soil samples were taken and dried completely in an oven. Then, the original soil moisture percentage (*w*/*w*) was calculated based on the weight of the completely dried soil. We controlled the soil water via daily spraying and created four different soil moisture contents: 5%, 10%, 15%, and 20% (*w*/*w*). Four containers (two pots for control and priming, respectively) were prepared for each soil moisture condition. Ten seeds were sown at 1 cm under the soil surface and 270 seeds (27 genotypes × 10 seeds) were sown in one container. Plant emergence was studied using the mean plant emergence time (MET), the time to achieve 50% plant emergence (E50), and plant emergence uniformity (EU). MET and E50 were calculated in the same way as MGT and G50. EU was calculated as the standard deviation of MET, based on the following formula [19].

(1) $$S_{t}\;(\mathrm{EU})\;=\;\sqrt{\sum_{i=1}^{k}n_{i}{\left(t_{i}-\bar{t}\right)}^{2}/\left(\sum_{i=1}^{k}n_{i}-1\right)}$$

where $\bar{t}$ is the mean germination(emergence) time; *t_i_* is the time between the start of the experiment and the *i*th observation (day or hour); *n_i_* is the number of seeds germinated (emerged) in the time *i*, and *k* is the last time of germination.

We measured plant height (PH) of all plants at seven days after planting and calculated plant height uniformity (PHU) as the coefficient of variation between the plants we observed. We conducted cluster analysis and principal component analysis using JMP version 13 (SAS Institute, Inc., Cary, NC, USA) We calculated statistical differences using a *t*-test.

## 3. Results

### 3.1. Cluster Analysis

**Figure 1:**
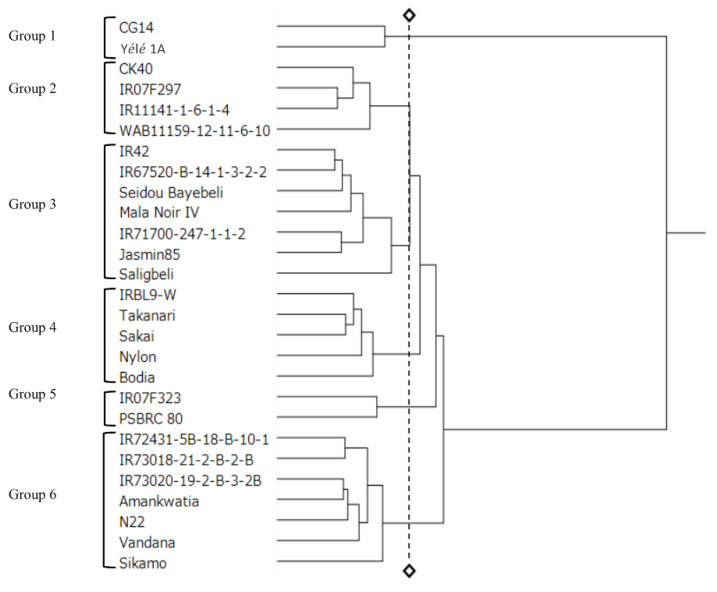
Result of cluster analysis of the 27 rice genotypes based on time to achieve 50% germination (G50), mean germination time (MGT), time to achieve 50% plant emergence (E50), mean plant emergence time (MET), plant emergence uniformity (EU), plant height (PH) and plant height uniformity (PHU) in 4 soil moisture conditions (5%, 10%, 15% and 20%) by hierarchical cluster analysis.

We carried out a hierarchical cluster analysis based on G50, MGT, E50, MET, plant EU, PH, and PHU under four soil moisture conditions (5%, 10%, 15%, and 20%). Cluster analysis divided the 27 genotypes into six groups (Figure 1). All the *O. glaberrima* were included in either Group 1 (G1) or Group 3 (G3), and G1 consisted entirely of *O. glaberrima*. Group 2 (G2) had three genotypes of *O. sativa* and one NERICA genotype. Group 4 (G4) contained five genotypes from four different origin countries: the Philippines, Japan, Ghana, and Guinea. Group 5 (G5) contained two genotypes from the Philippines. G2 and G5 include genotypes that have anaerobic germination (AG) + *Sub1* (Submergence 1) gene (IR07F292, IR07F323). Group 6 (G6) consisted of seven genotypes: two upland genotypes from India, two genotypes originating from Ghana, and three genotypes from the Philippines.

### 3.2. Germination

The MGT of the control seed was 51.8, 33.9, 38.5, 37.2, 37.0, and 31.0 in G1, G2, G3, G4, G5, and G6, respectively (Table 2). The MGT of the primed seed was 29.9, 24.5, 27.8, 25.5, 15.6, and 20.5 in G1, G2, G3, G4, G5, and G6, respectively. The MGT of the primed seed was significantly shorter than that of the control seed in all groups according to the *t*-test (*p* < 0.05). G50 showed the same tendency as MGT, and the G50 of primed seeds was significantly shorter than that of the control in all groups except G5. In G1, germination took longer for both primed and control seeds.

### 3.3. Plant Emergence

The E50 of the primed seeds tended to be shorter than that of the control seeds except under the 5% soil moisture condition (Table 3). There was an obvious priming effect in terms of more rapid plant emergence under the 10% soil moisture condition across the groups. Under the 10% soil moisture condition, the E50 of control seed was 146.0, 101.8, 102.4, 102.4, 98.1, 113.3, and 99.7 in G1, G2, G3, G4, G5, and G6, respectively, and the E50 of primed seeds was 81.5, 92.2, 86.5, 104.6, and 85.1 in G2, G3, G4, G5, and G6 respectively. In G1, the primed seed did not reach E50. There was a significant difference between control seeds and primed seeds in respect of E50, apart from in G1 and G5 under the 10% soil moisture condition. Under the 15% and the 20% soil moisture conditions, the ratio (priming/control) of E50 tended to be less than 1.00, but there were no significant differences, apart from in G6 under the 15% soil moisture condition. A priming effect on E50 was not found under the 5% soil moisture condition. With the exception of G1, the MET of primed seeds seemed to be more rapid than that of control seeds under 10% and 15% soil moisture conditions. In G3, G4, and G6, the MET of primed seeds was significantly shorter than was that of control seeds under the 10% soil moisture condition. Furthermore, in G2, G3, and G6, the MET of primed seeds was significantly shorter than that of control seeds under the 15% soil moisture condition. Conversely, under the 5% soil moisture condition, the MET of primed seeds appear to be larger than the control, and a significantly negative impact was found in G6. Under the 20% soil moisture condition, there was no significant difference, and the ratio (priming/control) of MET was 0.87, 0.91, 1.01, 1.03, 0.92, and 1.00 in G1, G2, G3, G4, G5, and G6, respectively. Compared with other parameters, EU did not show a clear tendency. There was a positive effect of priming under the 5% soil moisture condition, and there was a significant difference (*p* < 0.05) in the EU of G4 and G6.

### 3.4. Plant Height

In G2, G3, G4, and G6, the PH of primed seeds was significantly higher than that of the control under the 10% soil moisture condition (Table 4). In G2, G3, and G6, the PH of primed seed was significantly higher than that of the control under the 15% soil moisture condition. A positive effect of priming was not found under the 5% and the 20% soil moisture conditions. Under the 5% soil moisture condition, the PH of primed seeds appeared to be shorter than that of control seeds, and there were significant differences in G3 and G4. Under the 10% and 15% soil conditions, the PHU of primed seeds seemed to be enhanced. We found a significant difference between the PHU of priming and control in G6 under the 10% soil moisture condition and in G1, G2, and G3 under the 15% soil moisture condition (Table 4).

### 3.5. Correlation between Control and Priming

**Figure 2:**
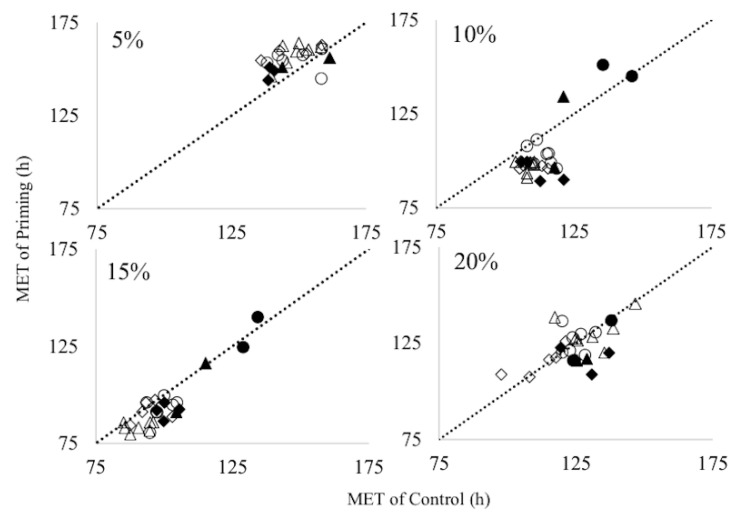
Relationship between control and priming of mean emergence time (MET) in the four soil moisture conditions. The dotted line indicates position of the x value equal to y value. Each plot shows one condition. Black circles, black rhombuses, white circles, white rhombus, black triangles and white triangles indicate genotype group1, 2, 3, 4, 5 and 6, respectively. Genotype groups are determined by cluster analysis (illustrated in Figure 2).

In respect of E50 and MET, we found a strongly significant positive correlation (*p* < 0.01) between control and primed seeds under the 10% and 15% soil moisture conditions (Table 5, Figure 2). Under the 20% soil moisture condition, there was a significant positive correlation between E50 and MET, though the coefficient of correlation was small. We did not find a significant correlation between control and primed seeds in respect to EU under all soil moisture conditions. There was a strongly significant positive correlation (*p* < 0.01) between control and primed seeds in respect to PH, apart from under the 20% soil moisture condition (Table 5). There was a significant positive correlation of PHU under the 10% (*p* < 0.05) and the 15% (*p* < 0.01) soil moisture conditions. Regarding the correlation diagram of MET, most of the plots were above the dotted line under the 5% soil moisture condition, suggesting that emergence takes much longer for primed seeds than for control seeds (Figure 2). Under the 10% and the 15% soil moisture conditions, most of the plots were below the dotted line, suggesting that emergence was faster for primed seeds than for control seed. Under the 20% soil moisture condition, some plots were on the dotted line and others were either above or below the line, suggesting there are genotypic differences in respect of priming efficacy.

### 3.6. Principal Component Analysis for Emergence

**Figure 3:**
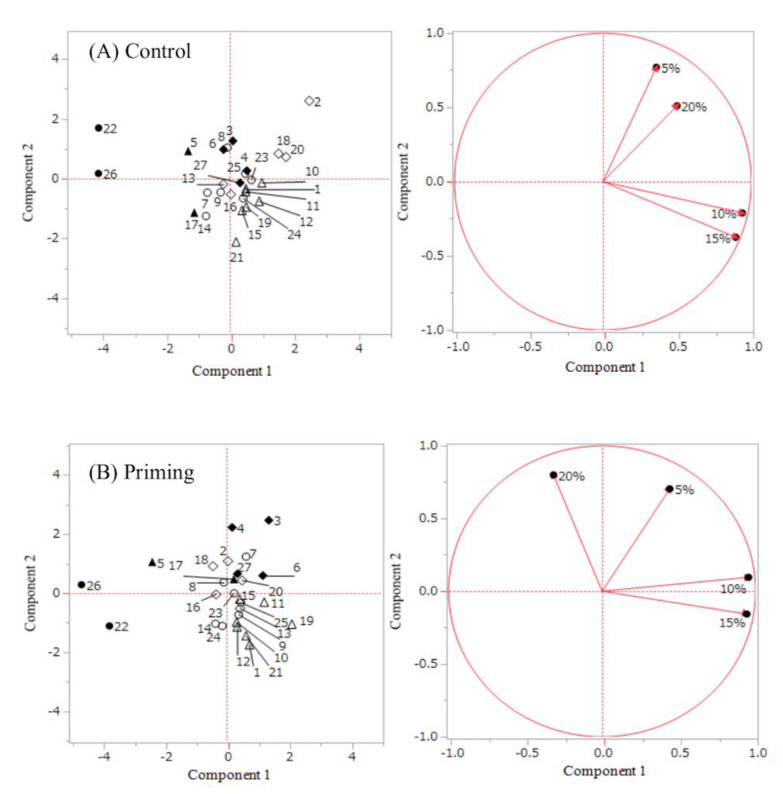
Results of principle component analysis of 27 genotypes based on mean emergence time (MET). (**A**) and (**B**) shows result of control and priming, respectively. Component 1 and 2 of (**A**) accounted for 51.5 and 25.9, respectively. Component 1 and 2 of (**B**) accounted for 52.2 and 29.2, respectively. MET was modified to negative values and analyzed. Each number shows one genotype (genotype numbers are illustrated in Table 1). Black circles, black rhombuses, white circles, white rhombus, black triangles and white triangles indicate genotype group1, 2, 3, 4, 5 and 6, respectively. Genotype groups are determined by cluster analysis (illustrated in Figure 1).

Figure 3A,B shows the results of principal component analysis based on the MET of control and primed seeds under the four soil moisture conditions. The symbols in the figures show the genotype groupings determined by cluster analysis (illustrated in Figure 1). G1 genotypes show a low score for component-1 both in control (Figure 3A) and primed seeds (Figure 3B), indicating that these genotypes have low plant emergence ability either with or without priming. Primed G2 genotypes were plotted near the 20% and 5% axis, suggesting that these genotypes have the potential to adapt to severe stress conditions via seed priming (Figure 3B). G3 was plotted near the center, either with or without priming (Figure 3A,B). G4 genotypes from control seeds were plotted near the 5% and 20% axis (Figure 3A) but those from primed seeds were plotted near the center (Figure 3B). G5 genotypes had a low score for component-1, but one genotype showed a high score for component-2. G6 genotypes were plotted near the 10% and 15% axis either with or without priming (Figure 3A,B).

## 4. Discussion

### 4.1. Growth Improvement Due to Seed Priming

Seed priming enhanced the parameters for germination (MGT and G50) significantly in all cluster groups, apart from the G50 of G5 (Table 2). Seed priming also appeared to enhance the growth parameters (MET, E50, and PH) in all groups apart from G1. Under the 10% soil moisture condition, priming improved the E50 and PH of G2, G3, G4, and G6 significantly (Table 3 and Table 4). The effect of priming on MET was similar to that on E50. Under the 15% soil moisture condition, there was a significant improvement in MET and PH in G2, G3, and G6 (Table 3 and Table 4). Matsushima and Sakagami (2013) [7] reported that priming efficacy was considerable under a moderately dry soil condition. Our findings are in accordance with this. Hydro priming increases α-amylase activity and total sugar content through soaking and drying the seed [6,12,21]. In primed seeds, significant correlations have been observed between α-amylase activity and mean emergence time, α-amylase activity and seedling dry weight, and soluble sugar content and seedling dry weight [11]. Moreover, priming helps maintain uniform growth, which is related to α-amylase activity and soluble sugar content [11]. Our previous study reported that seed priming increased PHU [16]. In this study, our findings demonstrate that priming treatment improved seed growth in almost all genotypes.

### 4.2. Priming Efficacy under Excessive Stress Conditions

Conversely, seed priming tends to restrict both emergence and plant growth under the 5% soil moisture condition. In G6, seed priming had significant negative impacts on MET and E50 under the 5% soil moisture condition (Table 3 and Table 4). There were also significant negative effects on PH in G3 and G4 under the 5% soil moisture condition (Table 4). Matsushima and Sakagami (2013) [7] reported that the effects of seed priming tend to be smaller under severe dry conditions (3% of VMC using sandy soil). Furthermore, Matsushima and Sakagami (2013) [7] suggested that seed priming promotes root growth compared to that of control seeds under declining soil moisture conditions. Even when there was no difference in emergence time between primed and control seeds, primed seeds grew better than control seeds after plant emergence [16]. In the current study, priming treatment improved only emergence uniformity significantly in G4 and G6 under the 5% soil moisture condition (Table 3), indicating that priming can have positive effects after emergence even in severely dry conditions. Under the 20% soil moisture condition, there were no significant positive differences between control and primed seeds in respect to all parameters in all groups. The effects of priming appear to diminish with increasing soil moisture content [7]. However, findings from previous research indicated that priming was effective even under high soil moisture conditions, such as anaerobic conditions, and that the efficacy differed according to plant genotype [17,18]. Regarding flooding conditions, Ella et al. (2011) [17] reported that the enhanced features of primed seeds are accounted for by scavenging reactive oxygen species (ROS) and mobilized carbohydrates. Superoxide dismutase and catalase activity are important for scavenging ROS, and Ella et al. (2011) [17] showed that seed priming increased the activity of these enzymes clearly in anaerobic resistant genotypes. In our study, the MET and E50 of primed seeds appeared to be earlier in G2 and G5 than in other groups under the 20% soil moisture condition (Table 3). In G2 and G5, the ratio (priming/control) was less than 1.00. Further, in principal component analysis, primed G2 and G5 genotypes were plotted near the 20% axis of MET (Figure 3B), indicating that these genotypes have the potential for adaption to wet conditions through using seed priming. G2 and G5 include genotypes that have anaerobic germination (AG) + *Sub1* (Submergence 1) gene (IR07F292, IR07F323). The genotypes in these groups were developed for AG and submergence tolerance (*Sub1*) and are characterized by rapid germination even when submerged [22]. Under submerged conditions, these genotypes produce a long coleoptile due to high α-amylase activity and high sucrose and glucose concentration in germinating seeds [23]. Our findings indicate that the anaerobic-tolerant genotypes (IR07F292, IR07F323) in G2 and G5 tend to exhibit priming efficacy under 20% soil moisture conditions.

### 4.3. Genotypic Difference of Plant Growth under Several Different Soil Moisture Conditions

Regarding MET, E50, PH, and PHU, we found a significant positive correlation between control and primed seeds under the 10% and 15% soil moisture conditions (Table 5). Meanwhile, under the 5% and 20% soil moisture conditions, either the coefficient of correlation between control and primed seeds was small or significant correlations were not found (Table 5, Figure 2), indicating that the properties shown by primed seeds under 5% and 20% soil moisture conditions are different to those of control seeds. Moreover, these findings indicate that there is a genotypic difference in seed priming efficacy and that this difference increases under wet conditions. G1 included two *O. glaberrima* genotypes (CG14, Yélé 1A). In this group, we did not find significant differences between control and primed seeds in respect of all parameters and under all soil moisture conditions. G1 seeds took much longer to emerge, and the subsequent PH was shortest under the 10%, 15%, and 20% soil moisture conditions (Table 3 and Table 4). Hence, G1 genotypes showed a low score for component-1 for both control and primed seeds (Figure 3A,B), suggesting that they have low emergence ability either with or without priming. The other three *O. glaberrima* genotypes (Seidou Bayebeli, Mala Noir IV, and Saligbeli) were included in G3, and the MET and PH of G3 were enhanced significantly under the 10% and 15% soil moisture conditions. These findings for G1 and G3 indicated that *O. glaberrima* has differences in reactivity to seed priming. G5 genotypes also showed a low component-1 in both control and priming, suggesting that they have low emergence ability either with or without priming. However, the IR07F323 of primed seeds was plotted near the 20% axis of MET (Figure 3B). Primed seeds of G2 genotypes were plotted near the 20% and 5% axis of MET (Figure 3B), suggesting that seed priming of G2 genotypes provides the potential for adaptive emergence under 5% and 20% soil moisture conditions. As mentioned above, G2 and G5 include AG + *Sub1* genotypes that have the potential to adapt well to wet conditions. The initial growth of anaerobic-tolerant genotypes under flooded conditions is accelerated by priming [17]. This study shows that G2 and G5, which include AG genotypes, have the potential for good adaption to wet conditions and that their growth might be accelerated by seed priming. Control of G4 showed the highest PH and earliest E50 and MET under the 20% soil moisture condition (Table 3 and Table 4), suggesting that G4 genotypes adapt well to wet conditions, as do G2 and G5 genotypes. Conversely, the ratios (priming/control) of PH, E50, and MET were 0.82, 0.98, and 1.03, respectively, under the 20% soil moisture condition. Additionally, the results of the principal component analysis show that G4 genotypes from control seeds were plotted near the 5% and 20% axis of MET (Figure 3A), whereas G4 genotypes from primed seeds were plotted near the center (Figure 3B), suggesting that G4 genotypes showed good initial growth under wet conditions, but seed priming did not accelerate their growth features under wet conditions. G6 included all upland genotypes of *O. sativa* (N22 and Vandana). Regarding the control seeds of G6, PH was the highest, and E50 and MET were the shortest under the 15% soil moisture condition. Furthermore, seed priming accelerated PH, E50, and MET significantly under the 10% and 15% soil moisture conditions (Table 3 and Table 4). Additionally, these G6 genotypes were plotted near the 10% and 15% axis of MET either with or without priming, suggesting that they adapted well to the moderate dry condition of 10% and 15% soil moisture and that their characteristics showed good improvement.

## 5. Conclusions

This study compared the efficacy of hydro priming of seeds of 27 genotypes of rice under different soil moisture conditions. As with previous reports, seed priming appeared to improve the performance of rice seed sown directly under the moderate dry conditions of 10% and 15% soil moisture. However, priming efficacy was low under 5% and 20% water stress conditions. This study has identified wide genotypic differences in priming efficacy under a 20% soil moisture condition. In particular, two genotypic groups that include anaerobic-tolerant genotypes (IR07F292, IR07F323) tended to show priming efficacy under 20% soil moisture conditions. Conversely, one genotypic group showed good initial growth under wet conditions, but the growth features of the plants were not enhanced by seed priming under wet conditions. Furthermore, our study findings indicate that *O. glaberrima* genotypes have differences in reactivity to seed priming. Also, one genotypic group included all upland genotypes of *O. sativa* (N22 and Vandana), which adapt well to moderately dry conditions of 10% and 15% of soil moisture, and their growth features were improved greatly by seed priming.

## Figures and Tables

**Table 1:** List of 27 genotypes.

No.	Species	Genotype	Origin	Adaptation or Characters
1	*Oryza sativa*	Amankwatia	Ghana	Lowland and irrigated
2		Bodia	Ghana	Lowland and irrigated
3		CK40	Guinea	Lowland
4		IR 07F297	Philippines	Anaerobic germination, *Sub1*
5		IR 07F323	Philippines	Anaerobic germination, *Sub1*
6		IR 42	Philippines	Irrigated
7		IR11141-1-6-1-4	Philippines	Elongation
8		IR67520-B-14-1-3-2-2	Philippines	Submergence
9		IR71700-247-1-1-2	Philippines	Lowland
10		IR72431-5B-18-B-10-1	Philippines	Elongation
11		IR73018-21-2-B-2-B	Philippines	Submergence
12		IR73020-19-2-B-3-2B	Philippines	Submergence
13		IRBL9-W	Philippines	Blast resistance, *Sub1*
14		Jasmin85	Ghana	Lowland and irrigated
15		N 22	India	Upland
16		Nylon	Guinea	Deepwater
17		PSBRC 80	Philippines	Lowland
18		Sakai	Ghana	Lowland and irrigated
19		Sikamo	Ghana	Lowland and irrigated
20		Takanari	Japan	Irrigated
21		Vandana	India	Upland
22	*O. glaberrima*	CG14	Senegal	Lowland
23		Mala Noir IV	Niger	Deepwater
24		Saligbeli	Guinea	Deepwater
25		Séidou Bayebeli	Guinea	Upland
26		Yélé 1A	Mali	Deepwater
27	Interspecific progeny (NERICA)	WAB1159-2-12-11-6-10	Guinea	Lowland

**Table 2:** Effects of priming on mean germination time (MGT) and time to achieve 50% germination (G50) of six genotypic groups.

	MGT (h)				G50 (h)			
	Control	Priming	Ratio	*t*-Test	Control	Priming	Ratio	*t*-Test
Group 1	51.8	29.9	0.58	**	60.0	28.9	0.48	**
Group 2	33.9	24.5	0.72	**	30.6	21.6	0.71	**
Group 3	38.5	27.8	0.72	**	37.8	25.0	0.66	**
Group 4	37.2	25.5	0.68	***	34.7	22.5	0.65	***
Group 5	37.0	15.6	0.42	*	34.3	12.0	0.35	ns
Group 6	31.0	20.5	0.66	**	28.8	17.3	0.60	**

Genotypic groups were determined by cluster analysis (illustrated in Figure 1), and the data are the means of genotypes. *, **, and *** indicate a significant difference (*p* < 0.05, *p* < 0.01, and *p* < 0.001, respectively), and “ns” indicates a non-significant difference between control and priming by a *t*-test.

**Table 3:** Effects of priming on time to achieve 50% plant emergence (E50), mean plant emergence time (MET), and plant emergence uniformity (EU) of six genotypic groups under four soil moisture conditions.

	Soil Moisture (*w*/*w*)															
	5%				10%				15%				20%			
	Control	Priming	Ratio	*t*-Test	Control	Priming	Ratio	*t*-Test	Control	Priming	Ratio	*t*-Test	Control	Priming	Ratio	*t*-Test
E50 (h)																
Group 1	-	-	-	-	146.0	-	-	-	127.5	-	-	-	-	-	-	-
Group 2	129.1	138.0	1.07	ns	101.8	81.5	0.80	*	86.3	79.3	0.92	ns	118.3	108.9	0.92	ns
Group 3	144.3	142.7	0.99	ns	102.4	92.2	0.90	*	85.5	80.8	0.94	ns	122.4	122.0	1.00	ns
Group 4	143.3	-	-	-	98.1	86.5	0.88	***	82.7	78.8	0.95	ns	112.5	109.9	0.98	ns
Group 5	149.9	-	-	-	113.3	104.6	0.92	ns	95.6	96.7	1.01	ns	119.9	104.8	0.87	ns
Group 6	147.1	152.3	1.04	*	99.7	85.1	0.85	***	79.5	72.3	0.91	*	134.7	121.1	0.90	ns
MET (h)																
Group 1	-	-	-	-	140.9	148.2	1.05	ns	131.6	132.3	1.00	ns	137.4	120.0	0.87	ns
Group 2	138.2	144.7	1.05	ns	112.1	94.6	0.84	ns	100.7	91.7	0.91	*	128.1	117.0	0.91	ns
Group 3	149.7	154.8	1.03	ns	113.8	103.0	0.90	*	98.7	92.7	0.94	*	124.8	126.4	1.01	ns
Group 4	143.4	159.0	1.11	ns	109.3	97.4	0.89	**	94.5	91.5	0.97	ns	112.0	115.3	1.03	ns
Group 5	152.7	153.4	1.01	ns	119.6	115.4	0.97	ns	109.9	103.7	0.94	ns	127.2	116.4	0.92	ns
Group 6	148.6	158.3	1.07	**	108.7	97.1	0.89	**	90.7	83.6	0.92	**	131.4	131.3	1.00	ns
EU																
Group 1	-	-	-	-	16.6	13.6	0.82	ns	20.9	21.0	1.01	ns	15.0	18.2	1.22	ns
Group 2	13.7	10.4	0.76	ns	12.7	7.8	0.61	*	21.7	12.0	0.55	*	20.7	14.4	0.70	ns
Group 3	11.4	9.0	0.79	ns	8.0	8.6	1.08	ns	12.9	9.8	0.76	ns	17.7	22.5	1.27	*
Group 4	18.1	5.3	0.29	*	14.7	7.0	0.47	*	15.5	13.2	0.85	ns	11.3	15.3	1.35	ns
Group 5	12.4	-	-	-	13.4	16.5	1.23	ns	20.2	13.3	0.66	ns	18.9	16.6	0.88	ns
Group 6	13.4	8.8	0.66	*	15.0	12.0	0.80	ns	12.2	12.0	0.99	ns	22.7	21.6	0.95	ns

Genotypic groups were determined by cluster analysis (illustrated in Figure 1), and the data are the means of genotypes. *, **, and *** indicate a significant difference (*p* < 0.05, *p* < 0.01, and *p* < 0.001, respectively), and “ns” indicates a non-significant difference between control and priming by a *t*-test. A bar indicates no data.

**Table 4:** Effects of priming on plant height (PH) and plant height uniformity (PHU) of six genotypic groups under four soil moisture conditions.

	Soil Moisture (*w*/*w*)															
	5%				10%				15%				20%			
	Control	Priming	Ratio	*t*-Test	Control	Priming	Ratio	*t*-Test	Control	Priming	Ratio	*t*-Test	Control	Priming	Ratio	*t*-Test
PH (cm)																
Group 1	-	-	-	-	2.4	2.7	1.12	ns	4.3	4.1	0.96	ns	1.9	2.1	1.12	ns
Group 2	2.9	2.4	0.84	ns	6.1	8.0	1.33	*	7.9	9.8	1.25	*	5.3	4.9	0.92	ns
Group 3	2.4	1.5	0.62	***	5.8	7.6	1.31	***	8.4	9.7	1.15	*	4.5	4.6	1.03	ns
Group 4	2.5	1.4	0.54	*	6.2	7.7	1.24	*	8.4	9.0	1.08	ns	5.6	4.6	0.82	ns
Group 5	1.6	0.9	0.52	ns	4.4	5.6	1.28	ns	6.5	7.1	1.09	ns	3.8	4.8	1.25	ns
Group 6	1.8	1.2	0.68	ns	6.1	8.7	1.43	**	9.0	10.3	1.15	**	4.0	4.9	1.22	ns
PHU																
Group 1	-	-	-	-	43.8	45.7	1.04	ns	66.3	56.0	0.84	*	53.4	49.1	0.92	ns
Group 2	38.3	43.9	1.15	ns	28.1	22.8	0.81	ns	41.2	15.7	0.38	*	31.3	31.0	0.99	ns
Group 3	45.2	53.3	1.18	*	27.3	24.5	0.90	ns	27.6	16.3	0.59	*	43.4	38.6	0.89	ns
Group 4	55.9	63.1	1.13	ns	26.0	20.9	0.80	ns	25.8	21.8	0.84	ns	23.5	34.5	1.47	ns
Group 5	49.8	56.7	1.14	ns	47.5	38.8	0.82	ns	38.4	23.5	0.61	ns	39.0	27.6	0.71	ns
Group 6	49.9	62.7	1.26	ns	32.4	20.7	0.64	*	18.6	12.4	0.67	ns	47.7	37.6	0.79	ns

Genotypic groups were determined by cluster analysis (illustrated in Figure 1), and data are the means of genotypes. *, **, and *** indicate significant difference (*p* < 0.05, *p* < 0.01, and *p* < 0.001, respectively), and “ns” indicates non-significant difference between control and priming by a *t*-test. A bar indicates no data.

**Table 5:** Correlation coefficients (r) of control and priming of the time to achieve 50% plant emergence (E50), mean plant emergence time (MET), plant emergence uniformity (EU), plant height (PH), and plant height uniformity (PHU) under four soil moisture conditions.

	Soil Moisture (*w/w*)			
	5%	10%	15%	20%
E50	0.290 ^ns^	0.754 **	0.814 **	0.490 *
MET	0.532 **	0.762 **	0.903 **	0.425 *
EU	0.327 ^ns^	0.257 ^ns^	0.201 ^ns^	0.277 ^ns^
PH	0.761 **	0.779 **	0.904 **	0.297 ^ns^
PHU	0.270 ^ns^	0.400 *	0.706 **	0.035 ^ns^

* and ** indicate that the correlation coefficient is significant (*p* < 0.05 and *p* < 0.01, respectively) and “ns” indicates non-significance.

## References

[1] Balasubramanian V., Sie M., Hijmans R.J., Otsuka K. (2007). Increasing Rice Production in Sub-Saharan Africa: Challenges and Opportunities. Adv. Agron..

[2] WARDA (Africa Rice Center) (2007). 2007 Africa Rice Trends.

[3] Somado E.A., Guei R.G., Keya S.O., WARDA (Africa Rice Center)/FAO/SAA (2008). NERICA®: The New Rice for Africa—A Compendium.

[4] Du L.V., Tuong T.P., Pandey S., Mortimer M., Wade L., Tuong T.P., Lopez K., Hardy B. (2002). Enhancing the Performance of Dry-Seeded Rice: Effect of Seed Priming, Seeding Rate, and Time of Seeding. Direct Seeding: Research Strategies and Opportunities, Proceedings of the International Workshop on Direct Seeding in Asian Rice Systems: Strategic Research Issues and Opportunities, Bangkok, Thailand, 25–28 January 2000.

[5] Farooq M., Siddique K.H.M., Rehman H., Aziz T., Lee D.J., Wahid A. (2011). Rice Direct Seeding: Experiences, Challenges and Opportunities. Soil Tillage Res..

[6] Farooq M., Barsa S.M.A., Wahid A. (2006). Priming of Field-Sown Rice Seed Enhances Germination, Seedling Establishment, Allometry and Yield. Plant Growth Regul..

[7] Matsushima K.-I., Sakagami J.-I. (2013). Effects of Seed Hydropriming on Germination and Seedling Vigor during Emergence of Rice under Different Soil Moisture Conditions. Am. J. Plant Sci..

[8] Khan A.A. (1992). Preplant Physiological Seed Conditioning. Hortic. Rev..

[9] Taylor A.G., Allen P.S., Bennett M.A., Bradford K.J., Burris J.S., Misra M.K. (1998). Seed Enhancements. Seed Sci. Res..

[10] Lee S.S., Kim J.H. (2000). Total Sugars, α-amylase Activity, and Germination after Priming of Normal and Aged Rise Seeds. Korean J. Crop Sci..

[11] Farooq M., Wahid A., Ahmad N., Asad S.A. (2010). Comparative Efficacy of Surface Drying and Re-Drying Seed Priming in Rice: Changes in Emergence, Seedling Growth and Associated Metabolic Events. Paddy Water Environ..

[12] Lee S.S., Kim J.H., Hong S.B., Kim M.K., Park E.H. (1998). Optimum Water Potential, Temperature, and Duration for Priming of Rice Seeds. Korean J. Crop Sci..

[13] Soltani E., Soltani A. (2015). Meta-Analysis of Seed Priming Effects on Seed Germination, Seedling Emergence and Crop Yield: Iranian Studies. Int. J. Plant Prod..

[14] Binang W.B., Shiyam J.O., Ntia J.D. (2012). Effect of Seed Priming Method on Agronomic Performance and Cost Effectiveness of Rainfed, Dry-Seeded NERICA Rice. Res. J. Seed Sci..

[15] Harris D., Pathan A.K., Gothkar P., Joshi A., Chivasa W., Nyamudeza P. (2001). On-Farm Seed Priming: Using Participatory Methods to Revive and Refine a Key Technology. Agric. Syst..

[16] Nakao Y., Asea G., Minoru Y., Nobuki K., Hiroyuki H., Kisho M., Yabuta S., Rieko K., Jun-Ichi S. (2018). Development of Hydropriming Techniques for Sowing Seeds of Upland Rice in Uganda. Am. J. Plant Sci..

[17] Ella E.S., Dionisio-Sese M.L., Ismail A.M. (2011). Seed Pre-Treatment in Rice Reduces Damage, Enhances Carbohydrate Mobilization and Improves Emergence and Seedling Establishment under Flooded Conditions. AoB Plants.

[18] Illangakoon T.K., Ella E.S., Ismail A.M., Marambe B., Keerthisena R.S.K., Bentota A.P., Kulatunge S. (2016). Impact of Variety and Seed Priming on Anaerobic Germination-Tolerance of Rice (Oryza sativa L.) Varieties in Sri Lanka. Trop. Agric. Res..

[19] Ranal M.A., De Santana D.G. (2006). How and Why to Measure the Germination Process?. Braz. J. Bot..

[20] Katsura K., Tsujimoto Y., Oda M., Matsushima K.I., Inusah B., Dogbe W., Sakagami J.I. (2016). Genotype-by-Environment Interaction Analysis of Rice (Oryza spp.) Yield in a Floodplain Ecosystem in West Africa. Eur. J. Agron..

[21] Ando H., Kobata T. (2002). Effect of Seed Hradening on the Seedling Emergence and α-amylase Activity in the Grains of Weat and Rice Sown in Dry Soil. Jpn. J. Crop Sci..

[22] EL-Hendawy S.E., Sone C., Ito O., Sakagami J.I. (2011). Evaluation of Germination Ability in Rice Seeds under Anaerobic Conditions by Cluster Analysis. Res. J. Seed Sci..

[23] Adachi Y., Sugiyama M., Sakagami J.I., Fukuda A., Ohe M., Watanabe H. (2015). Seed Germination and Coleoptile Growth of New Rice Lines Adapted to Hypoxic Conditions. Plant Prod. Sci..

